# ESPR Uroradiology Taskforce—imaging recommendations in paediatric uroradiology, part VIII: retrograde urethrography, imaging disorder of sexual development and imaging childhood testicular torsion

**DOI:** 10.1007/s00247-015-3452-3

**Published:** 2015-12-01

**Authors:** Michael Riccabona, Kassa Darge, Maria-Luisa Lobo, Lil-Sophie Ording-Muller, Thomas A. Augdal, Fred E. Avni, Johan Blickman, Beatrice M. Damasio, Aikaterini Ntoulia, Frederika Papadopoulou, Pierre-Hughes Vivier, Ulrich Willi

**Affiliations:** Department of Radiology, Division of Pediatric Radiology, University Hospital LKH Graz, Auenbruggerplatz 34, 8036 Graz, Austria; Department of Radiology, The Children’s Hospital of Philadelphia, University of Pennsylvania, Philadelphia, PA USA; Department of Radiology, Hospital de Santa Maria-CHLN, University Hospital, Lisbon, Portugal; Department of Radiology and Nuclear Medicine, Unit for Paediatric Radiology, Oslo University Hospital, Oslo, Norway; Department of Radiology, University Hospital of Northern Norway, Tromsø, Norway; Department of Pediatric Radiology, Jeanne de Flandre Hospital, CHRU de Lille, Lille Cedex, France; Department of Radiology, Golisano Children’s Hospital, Rochester, NY USA; Department of Radiology, G. Gaslini Institute, Genoa, Italy; Department of Radiology, The Children’s Hospital of Philadelphia, Philadelphia, PA USA; Pediatric Ultrasound Center, Thessaloniki, Greece; Hôpital Privé de l’Estuaire, Radiologie, Le Havre, France; Department of Radiology, Kantonsspital Baden AG, Baden, Switzerland

**Keywords:** Urethra, Stricture, Fistula, Diverticulum, Congenital abnormality, Disorder of sexual development, Testicular torsion, Diagnostic imaging, Child

## Abstract

Three new consensus-based recommendations of the European Society of Paediatric Radiology Uroradiology Taskforce and the European Society of Urogenital Radiology Paediatric Working Group on paediatric uroradiology are presented. One deals with indications and technique for retrograde urethrography, one with imaging in the work-up for disorders of sexual development and one with imaging workflow in suspected testicular torsion. The latter is subdivided to suggest a distinct algorithm to deal with testicular torsion in neonates. These proposals aim to outline effective imaging algorithms to optimise diagnostic accuracy and to harmonize diagnostic imaging among institutions and practitioners.

## Introduction

The European Society of Paediatric Radiology (ESPR) Uroradiology Taskforce, in corporation with the European Society of Urogenital Radiology (ESUR) Paediatric Working Group, set out to provide recommendations and standards for imaging in paediatric uroradiology. The aim is to harmonize and optimize imaging techniques in children. Procedural recommendations and imaging algorithms for typical queries should allow for answering the relevant clinical and therapeutic questions at lowest possible radiation burden and by avoiding unnecessary procedures. Such measures should also create an environment where multi-institutional research and meta-analysis can be more easily performed and eventually should lead to more evidence by optimized imaging in neonates, infants and children.

Many procedures and clinical queries have been addressed in the existing recommendations of the task force and working group. We have addressed some of the few remaining entities and procedures, e.g., retrograde urethrography, which is a less commonly performed procedure today but still should be carried out in a standardized fashion on well-selected patients. The group also recommends an imaging algorithm in childhood testicular torsion.

The recommendations are again based on a thorough literature search and discussion among the group members. Consulted experts in the field included referring paediatric urologists, paediatric surgeons, paediatricians and paediatric radiologists. As with many conditions in childhood, limited evidence is available in the literature for some of these topics. Thus, existing recommendations are only in part evidence based; however, there are consensus statements from the ESPR and ESUR groups. The present proposals were discussed within the group via e-mail and internal group meetings. They were presented at the ESPR annual meeting in 2014 and discussed in public. All comments and suggestions were integrated in the elaboration of the proposed recommendations.

## Retrograde urethrography

Retrograde urethrography is an infrequent procedure and may be performed by the paediatric urologist rather than by the paediatric radiologist. For some indications, a careful technique is essential to proper demonstration of the urethra and its abnormality. Retrograde urethrography may be indispensable when antegrade urethrography is technically difficult or impossible and a suspected abnormality must be shown or excluded. This procedure is almost exclusively used in males.

Typical indications are post-traumatic injury, e.g., rupture and discontinuity as well as iatrogenic sequelae. Urethral stricture may develop after reconstructive surgery and improper or prolonged catheterisation (the latter particularly after cardiac surgery). Urethral narrowing occurs in rare congenital malformation, including urethral duplication, fistulae and prostatic utricle (possibly associated with hypospadias).

### Technique

**Fig. 1:**
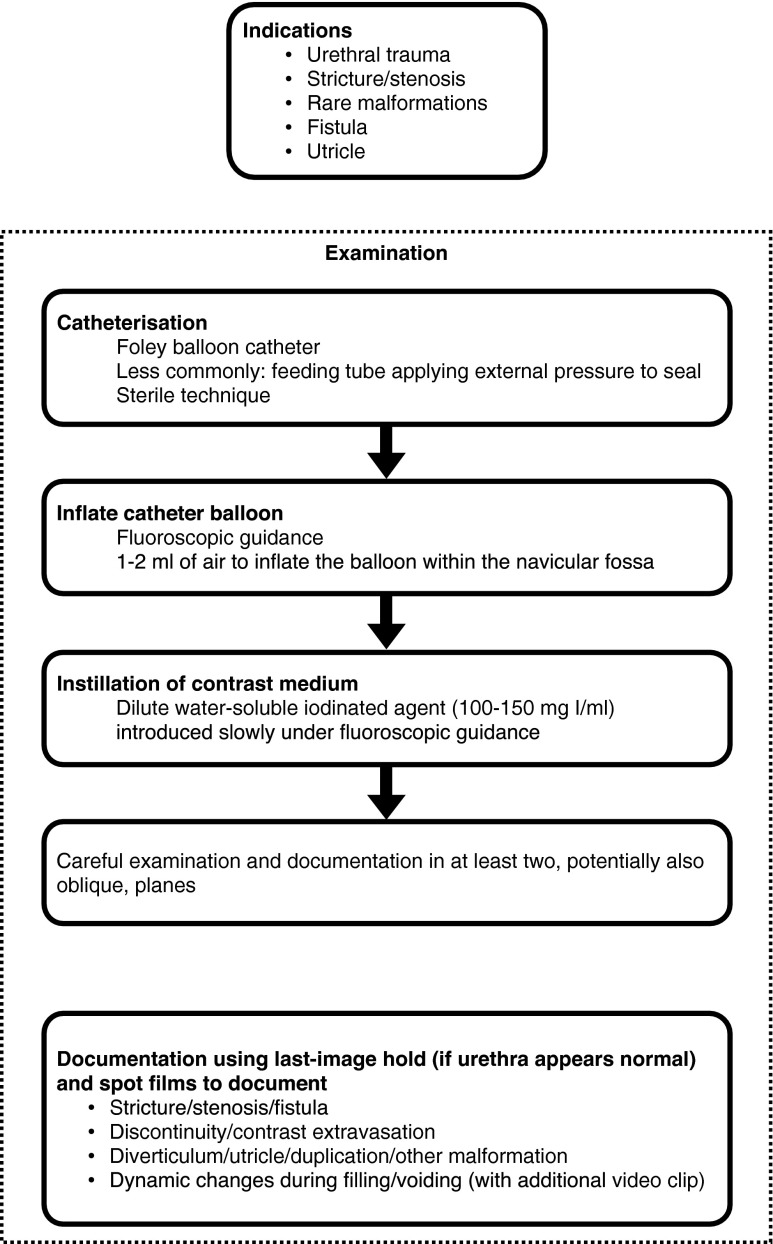
Suggestion on how to perform retrograde urethrography in children

At retrograde contrast filling of the urethra, contrast leakage must be withheld by proper occlusion of the external orifice for optimal distension and visualisation of the urethra. This requires a specific catheterisation technique, using a Foley catheter one size larger than age appropriate, with the balloon placed in the navicular fossa (this may be supported by gentle manual compression of the urethral orifice). After surgery with a urethral catheter in place, an additional small feeding tube may allow visualisation of the urethra before removing the surgically placed catheter. This is known as retrograde pericatheter urethrography. The contrast material is iso-osmolar, as used for voiding cystourethrography, i.e. radiopaque contrast concentration of 100 to 200 mg I/ml. After properly positioning the catheter under fluoroscopy guidance, the balloon is carefully inflated with just enough air necessary to prevent contrast leakage. Excessive inflation should be avoided. The urethra is then filled with the contrast agent using a syringe and carefully timed constant fluoroscopic monitoring. Anterior-posterior as well as lateral views are acquired (either by patient positioning or by rotating the tube) for detailed anatomical assessment. For documentation, spot films may become necessary instead of simple last image hold frames (Fig. 1). Testicular protection capsules must be avoided to prevent obscuring of potentially essential anatomical parts.

The retrograde technique can be used in combination with antegrade urethrography in selected patients. After performing the retrograde procedure, it may become necessary to advance the catheter into the bladder and complete the investigation with a conventional voiding cystourethrography, which includes antegrade urethrography during voiding. In the presence of a suprapubic catheter, simultaneous retrograde and antegrade urethrography may be possible, as required to delineate the exact length of the urethral pathology.

An alternative to fluoroscopic retrograde urethrography is the US-guided retrograde filling and distension of the urethra (a sonographic urethrogram) with saline (or US contrast agent) using the same technique of retrograde filling and distension of the urethra. It requires high-resolution linear transducers and a penile and transperineal approach.

## Imaging in disorders of sexual development

*Intersex*, now universally named *disorder of sexual development*, is another rare condition but with essential implications for the affected individual and their families. The prevalence at birth is 1:5,000 for complex anomalies possibly related to genital ambiguity. There is a markedly heterogeneous group of conditions: Identical genetic defects may result in various clinical presentations, and a similar phenotype may be the end point of different genetic pathways. Usually the query arises after birth when ambiguous outer genitalia are noted. Sometimes it is discovered incidentally during an investigation for other reasons such as urinary tract infection. A group of patients may become manifest because of growth or pubertal disorders. The mainstay of diagnosis is genetic and laboratory (endocrine) tests. Clinical inspection and history are of utmost importance.

The task of imaging is to determine the absence or presence of the inner genitalia to decide whether gonadal structures are male or female or, possibly, streak gonads, to offer information necessary for potential corrective surgery, and to evaluate for associated malformations and findings. In all age groups, the first imaging step usually is US, which in most situations can answer the relevant questions:

1. Is the uterus present or absent? The answer is most helpful. It is easier to obtain in the neonatal period because of the prominent uterine volume commonly seen during the first months after birth due to previous maternal hormonal stimulation. Also: Is there a vagina?
2. Are there gonads? Does their structural appearance suggest a male or a female phenotype?
3. Are there any associated malformations of the genital or urinary tracts? Include documentation of the adrenal appearance.

By using a transabdominal and a transperineal approach as well as US genitography, most of these questions can be answered sonographically. Appropriate recommendations have been issued.

However, in some situations, US is insufficient, particularly if access is problematic as to gonadal structures in an unusual position or in marked gonadal hypodysplasia. In these circumstances, particularly in older patients, MRI of the pelvis using high-resolution sequences may be very helpful. However, even with MRI, some structures may remain unclear; if tubular characteristics are suspected, the filling with saline or diluted contrast (as with genitography) may be necessary for defining their provenance. For detailed anatomical assessment of the inner genitalia, particularly in vaginal malformations with potential fistula, fluoroscopic genitography is still a recommended complementary tool. This could be performed with the same catheters in place as used for US genitography.

In complex hormonal disorders in older children, MRI of the adrenal glands and the brain as well as US to assess the absence or presence of breast tissue may be considered.

**Fig. 2:**
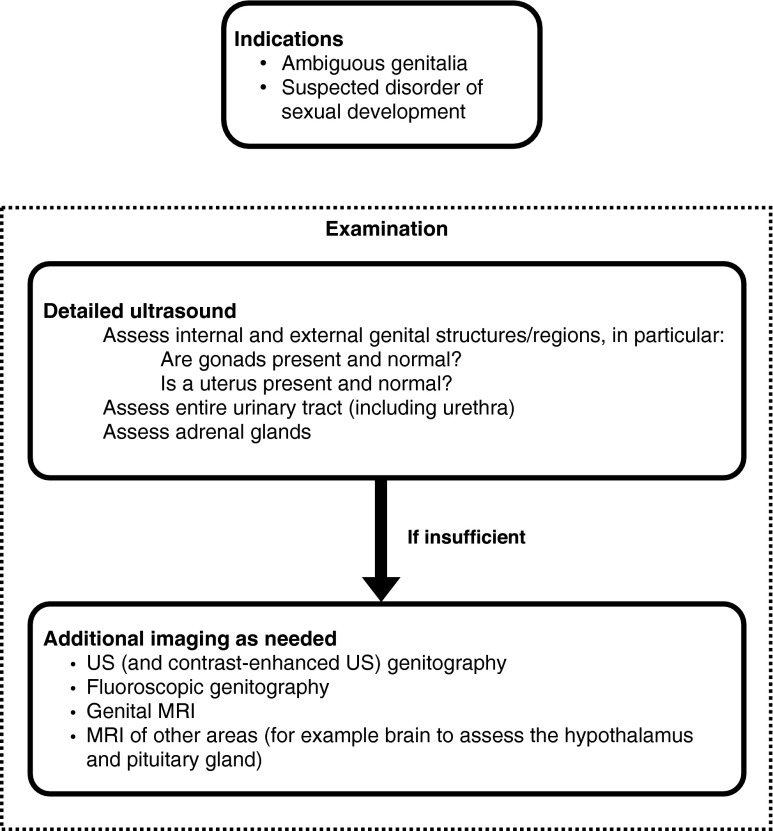
Recommendation for diagnostic imaging in children with disorder of sexual development

In general, these queries should be assessed by a highly specialised multidisciplinary team in a dedicated centre with efficient communication among all parties involved. Imaging should be tailored according to the individual situation, with the lowest possible invasiveness and after thorough discussion with the referring physician(s). The presented algorithm (Fig. 2) is proposed as a guide to decide about the necessary or unnecessary imaging steps.

## Imaging in childhood testicular torsion

There are two age-dependent peaks for testicular torsion: perinatally (within the first month of life; usually occurring prenatally or during birth) and in older children. The real perinatal incidence is unknown but is estimated at around 6 in 100,000 births. Early torsion usually is extravaginal and the overall salvage rate is approximately 9%, approaching zero if the event was prenatal. There are, however, indications that emergency detorsion yields salvage rates around 40%. The second peak, in older children, has two different presentations: either during early childhood (older than 1 month of age) or during puberty; the latter is the most common manifestation with the majority of patients older than 10 years. Its true incidence is unknown, but is estimated at 4 per 100,000 for boys younger than 18 years; in almost all cases, it is an intravaginal torsion based on the “bell clapper” appearance. Mesorchial torsion is rather rare. Furthermore, it is more common in undescended testes and then may present as an inguinal mass. In comparison with perinatal testicular torsion, the salvage rate is much higher, in general higher than 60% with one report quoting a 94% salvage rate.

The natural history of torsion is either spontaneous detorsion or progressive tissue injury due to ischemia and congestion—2 h after the event there is damage to spermatogenesis, 4 to 6 h thereafter spermatogenesis is likely destroyed. Eight hours later, the endocrine function is damaged as well, and after 10 to 12 h there is complete testicular necrosis. However, time is only one aspect; another is the degree of torsion—partial torsion is far less threatening and allows for a longer window of time than complete or multiple torsions. In testicular torsion after the neonatal period, the treatment usually consists of prompt surgical exploration with detorsion and orchidopexy. If perfusion is not restored after manual detorsion and the testicle is necrotic, this testicle cannot be salvaged and orchiectomy must be performed with strongly recommended contralateral orchidopexy.

There are institutions and situations where (partial) manual detorsion under US guidance is performed, mostly by surgeons and urologists. This is controversial but may help to increase the salvage rate by shortening the duration of decreased perfusion; however, it will not replace emergency surgery. As complication rates of surgery are very low and—if these occur—usually not severe, there is a low threshold for going to surgery. This is reflected by the European Association of Urology Guidelines 2013 for suspected testicular torsion after the neonatal period, particularly as clinical symptoms remain unspecific.

Imaging by US should only be performed in equivocal cases. Practically speaking, US is often done mainly to confirm epididymo-orchitis when the clinical likelihood of torsion is low. The study must be performed without any delay using a high-frequency, high-resolution linear transducer. Colour Doppler studies with duplex spectral analysis of the testicular parenchyma and vessels are mandatory. Bilateral assessment is standard, for comparison. If performed by experienced investigators, US is a reliable diagnostic method. However, in doubtful cases, immediate surgical exploration is still mandatory. Only if testicular torsion can be ruled out with certainty, and/or other pathology causing the symptoms can be defined, surgery may be postponed. Testicular torsion can be ruled out if a normal spermatic cord (homogenous, linear and without thickening) and symmetrical, normal testicles (both parenchyma and perfusion) are seen on US. Additionally, US can often help to find another entity explaining the clinical symptoms (i.e. epididymo-orchitis).

Nuclear medicine studies and MRI with perfusion- and diffusion-weighted imaging are methods that may theoretically be useful in testicular torsion; however, these methods are normally not readily available and will most often cause diagnostic delay, hence are not applicable in the acute setting.

**Fig. 3:**
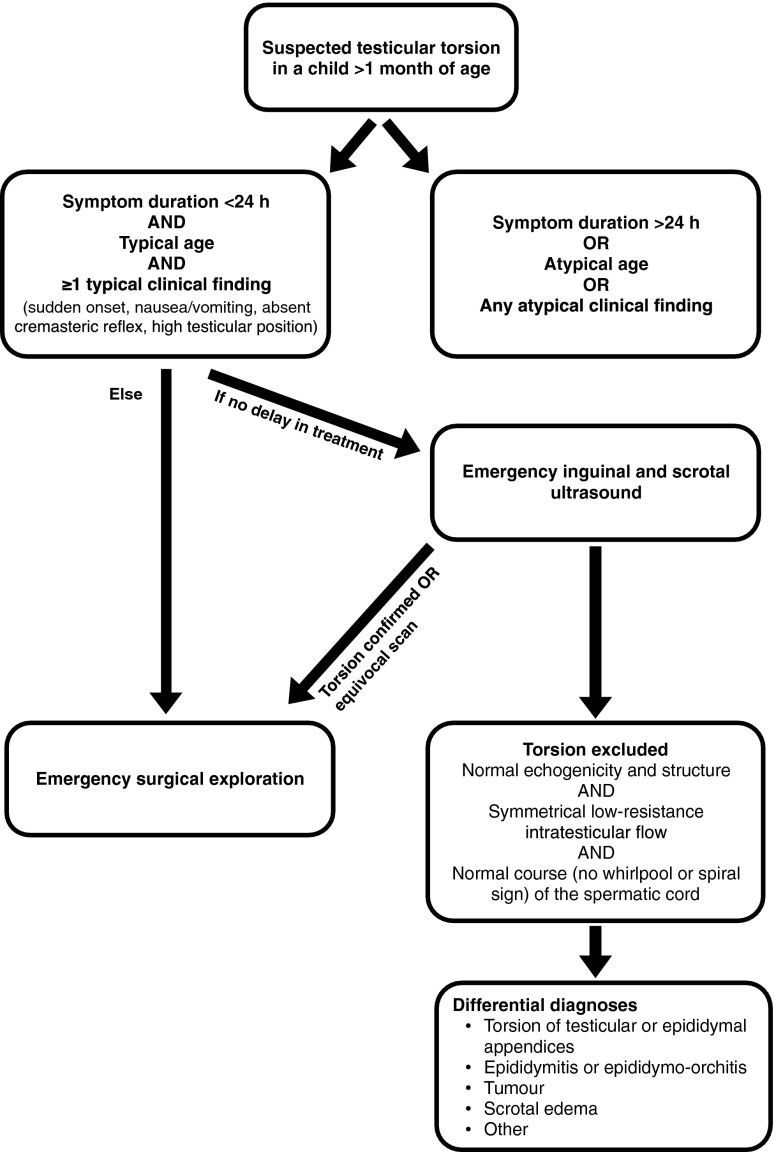
Recommended imaging of suspected testicular torsion beyond the neonatal age

Taking all aspects into account, the imaging algorithm for childhood testicular torsion (Fig. 3) is quite simple. In suspected testicular torsion (after the perinatal period), immediate surgical exploration is advocated when the clinical findings are highly suspicious. In equivocal cases, immediate US should be performed without diagnostic delay by an experienced ultrasonographer. MRI may be an option for complementing the assessment of differential diagnoses such as testicular tumour or undescended testes.

**Fig. 4:**
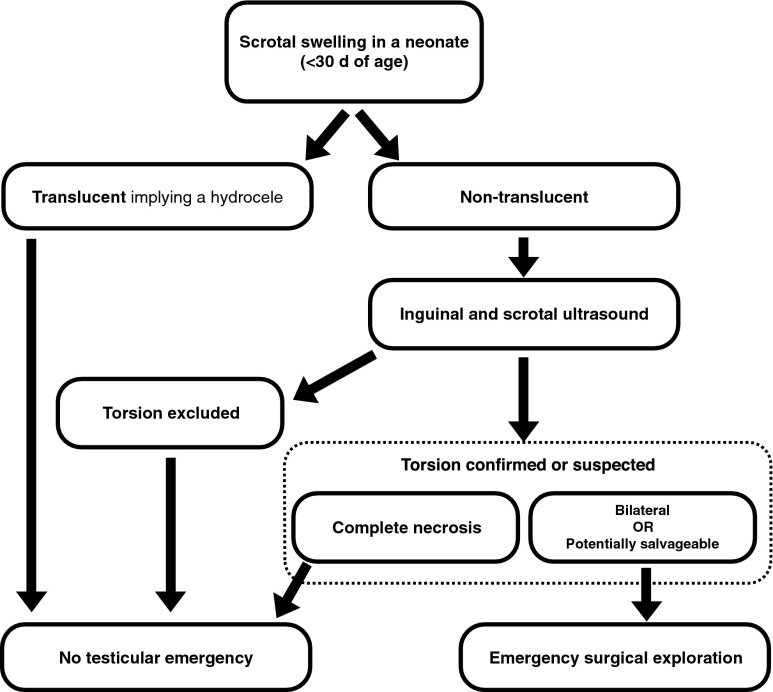
Recommendation for imaging of suspected testicular torsion in neonates

The algorithm in neonatal torsion (Fig. 4) is slightly different and more controversial. Because of the low prevalence of neonatal torsion there is practically no scientific evidence available regarding imaging and its role in the management of this condition. In neonatal torsion, the testicle is rarely salvageable, except in a few circumstances (e.g., partial torsion or if the torsion occurred during or just after birth and treatment was undertaken immediately). US examination followed by early surgical exploration has therefore become the most generally accepted approach for all cases with non-translucent scrotal swelling. In neonatal torsion, the testicle is often firm on palpation, with some bluish discolouration and lack of a cremasteric reflex. There may be some tenderness or a retracted testicle, but the patient is often asymptomatic. The US examination should be performed with a high-frequency linear transducer (12–18 MHz). The grey-scale findings are the most important because the colour Doppler assessment of a neonatal testicle can be very difficult even when the perfusion is normal. If US demonstrates a unilateral necrotic testis, it may be removed with elective surgery and a contralateral orchidopexy performed simultaneously. If there is bilateral involvement (with reportedly one-third being asynchronous), an immediate exploration is considered justified giving the risk of complete anorchia. In the rare occasion of unilateral disease with US signs of possible viable tissue, based on echogenicity and perfusion of the affected testicle, emergency surgery is also performed. Thus, immediate US should be performed when a neonate presents with non-translucent scrotal swelling, with stratification of further management depending on the US findings.

## Conclusion

Three new and important, though less frequent, conditions and related procedures were addressed in this taskforce session. Furthermore, comments and updates on the ongoing work, i.e. standardisation of terminology in pediatric uroradiology, were presented. The latter is not included in this report, as adaptations will have to be made after the feedback from all other subspecialties and groups involved.

The procedural recommendation for retrograde urethrography aims to standardize this infrequent investigation in order to attain optimal results at the lowest possible risk and may be of help to radiologists and institutions where this procedure is performed.

The imaging algorithm for queries in neonates, infants and children with ambiguous genitalia or suspected disorders of sexual development tries to include new terminology and definitions and highlights the clinically relevant information offered by imaging. Imaging is mainly based on detailed US, including US genitography. Fluorosopic genitography and MRI are complementary tools in selected cases based on the US finding and clinical necessities.

In the imaging algorithm for testicular torsion in childhood, we differentiate between the two main entities, the peri- and neonatal torsion and torsions in children and infants older than 1 month. Imaging should not cause any delay in diagnosis but contribute to increase the specificity of diagnosis of testicular torsion in order to avoid unnecessary surgical explorations.

It is the expectation of the group that based on standardisation of imaging and imaging algorithms, future meta-analysis or multicentre research can be facilitated and subsequent adaptations and updates of these proposals can be based on more solid evidence. Until this is available, the groups’ proposals will likely help to improve patient care by providing suggestions on how to proceed with these queries and examinations, still allowing for individual adaptation and respecting different local settings, options and needs. It also remains a task of paediatric radiology to make the most useful, economically feasible and radiation-sparing methods available to children. The establishment of a proper US service (reliably dealing with the relevant paediatric conditions including emergency settings 24 h a day and 7 days a week throughout the year) is an important action for granting proper health care to paediatric patients. At times, this is only achievable in cooperation with other (sub)specialties. As with all these proposals, the ESPR Uroradiology Taskforce and ESUR Paediatric Working Group heavily rely on feedback and input from everybody involved in the care of children.
